# Iontophoresis Improves the Impact on the Quality of Life of Children with Primary Hyperhidrosis—A Prospective Study and a Short Review

**DOI:** 10.3390/children11101253

**Published:** 2024-10-17

**Authors:** Florentina Nastase, Camelia Busila, Alin Codrut Nicolescu, Cristina Mihaela Marin, Alin Laurentiu Tatu

**Affiliations:** 1 Neuropsychomotor Rehabilitation Department, “Sf. Ioan” Emergency Clinical Hospital for Children, 800487 Galati, Romania; florentina34ro@yahoo.com; 2 Clinical Medical Department, Faculty of Medicine and Pharmacy, “Dunarea de Jos” University, 800008 Galati, Romania; cristina.marin@ugal.ro (C.M.M.); alin.tatu@ugal.ro (A.L.T.); 3 Pediatrics Department, “Sf. Ioan” Emergency Clinical Hospital for Children, 800487 Galati, Romania; 4 “Agrippa Ionescu” Emergency Clinical Hospital, 011773 Bucharest, Romania; 5 Multidisciplinary Integrated Center of Dermatological Interface Research MIC-DIR, “Dunărea de Jos” University, 800201 Galati, Romania; 6 Dermatology Department, “Sfanta Cuvioasa Parascheva” Hospital of Infectious Diseases, 800179 Galati, Romania

**Keywords:** hyperhidrosis, evaluation, quality of life, iontophoresis

## Abstract

Background: Primary hyperhidrosis (PH) is a somatic and idiopathic pediatric skin disease. The eccrine glands are tiny and very numerous, with approximately 3 million distributed throughout the skin. There is no commonly accepted amount of sweating to define hyperhidrosis, but people with this disease suffer real limitations integrating into society, which can be quantified through quality of life measurement scales. We want to draw attention to this disease and its impact on children’s quality of life because it is significant and there are no studies conducted on groups consisting solely of children. Methods: There are various quality of life evaluation questionnaires for hyperhidrosis. We studied 103 children with hyperhidrosis by monitoring their sweat severity and its impact on quality of life, using the Hyperhidrosis Disease Severity Scale. We compared the scale results before and after 10 days of iontophoresis. This study includes only children under 18 years old, treated with iontophoresis. Results: The average age of the group is 11.84 ± 2.89 years. Treatment success is recorded in 68 (66.02%) children, but a change in the score is recorded in 74 (71.84%) children. The average HDSS score at T0 is 2.95 ± 0.70, compared to the HDSS score at T1 of 1.92 ± 0.86. Conclusions: Hyperhidrosis has a negative impact on daily life, especially self-esteem, occupational productivity, emotional well-being, and interpersonal relationships. Iontophoresis is a safe and effective treatment method that reduces the severity of hyperhidrosis and increases the quality of life.

## 1. Introduction

Primary hyperhidrosis (PH) is a somatic and idiopathic pediatric skin disease, which can be inherited [1,2,3,4], characterized by significant sweating beyond physiological needs to maintain normal thermal regulation, which disappears during sleep [5,6,7,8]. Hyperhidrosis (HH) is classified as primary and secondary. Secondary hyperhidrosis occurs due to medication or a medical condition, affects older patients, and manifests as excessive generalized sweating during the day and night as well [9,10]. The onset of primary hyperhidrosis usually occurs in childhood and continues into adult life [2,11]. The genetic component of disease transmission is supported by the positive family history, even in the third generation [6,12,13]. It has been shown there are no pathological changes in the sweat glands [14]. Hyperhidrosis has a negative impact on daily life, especially patients’ self-esteem, occupational productivity, emotional well-being, and interpersonal relationships [15,16,17]. The concept of quality of life (QoL) first appeared in 1970 and was used as a synonym for self-esteem, life satisfaction, wellbeing, happiness, health, meaning of life, ability to take care of oneself, and functional independence [5]. Many diseases cause a decrease in the quality of life, especially skin diseases such as psoriasis, lichen planus, ulcers, acne, rosacea, cancers and infections, and inflammatory and degenerative diseases [18]. The World Health Organization defines the quality of life as the individual perception in comparison with one’s own expectations, and there are many influencing factors such as social standards, the values and culture of a country, and people’s goals [5].

Several treatment options are used for managing hyperhidrosis in the pediatric population. The first-line therapy is frequently the topical aluminum salt found in prescription antiperspirants. It is believed that the topical aluminum chloride mechanically obstructs the pores of the eccrine sweat glands and leads to the atrophy of the secretory cells. Oral anticholinergic use in the treatment of children with hyperhidrosis includes drugs such as glycopyrrolate and propantheline bromide. 

Electric current has been used since 1930 to introduce ions into the skin in a process known as iontophoresis, with tap water being the most common medium used. However, anticholinergic drugs can also be added to enhance efficacy. The effect is temporary and lasts up to 3 months [19]. 

Intradermal botulinum toxin injection produces a decrease in sweat production, inhibiting neurotransmission by affecting the nerve terminals. Toxins A and B are the two serotypes of botulinum toxin most commonly used. The major limiting factor for this treatment is the pain associated with injections. The options to help minimize this pain are cryotreatment and the application of topical anesthetics before the injection with botulinum toxin. Usually, the anhidrotic effect appears after three days of administration and can be maintained from 3 to more than 12 months [1,19]. 

For children with hyperhidrosis, surgical treatment may be a suitable option when the sweating is refractory to the less invasive treatments. Liposuction and curettage are used to only remove the glands from the axillae; the other areas affected by hyperhidrosis have contraindications for this procedure. Ultrasound is a minimally invasive treatment that uses the VASER System.

Video-assisted thoracoscopic sympathectomy consists of two to three incisions measuring less than 1 cm each, below the axillae, the lung is deflated, and a telescopic camera is introduced into the thoracic cavity. The sympathetic chain is located from T2—correlated with palmar hyperhidrosis—to T4—correlated with axillary hyperhidrosis. This treatment option has a short recovery time but may be associated with serious complications such as compensatory sweating, infection, pneumothorax, or hemothorax.

The therapeutic options for primary pediatric hyperhidrosis are available, but further studies in the pediatric population are needed to help guide appropriate management [19].

The main objective of this work was to determine the severity of hyperhidrosis via the completion of a Hyperhidrosis Disease Severity Scale (HDSS) questionnaire and determine its impact on the quality of life in children aged between 6 and 17, with the scale being applied before and after performing 10 iontophoresis sessions. This work can contribute to the understanding of this very complex disease and to the awareness of the marked effects it has on children’s development.

## 2. Materials and Methods

The study was initiated after the approval no. 31117/04.12.2023 of the Medical Council of the “Sf. Ioan” Emergency Clinical Hospital for Children, Galati, Romania. Informed consent was obtained from all subjects involved in the study, with content being expressed in simple terms and explained to the children and their parents throughout the research. Confidentiality principles were respected, the results being in the form of anonymized statistical data.

This is a prospective study conducted in the Department of Neuropsychomotor Rehabilitation of the hospital between 1 March and 31 May 2024. We had a group of 103 children in the study who were given to complete a Hyperhidrosis Disease Severity Scale questionnaire before the start of treatment—T0 and one week after its completion—T1.

The criteria to be included were: patients under 18 years of age at the time of consultation, who presented themselves at the clinic during the indicated period. The parents of the patients expressed their agreement to participate by signing the consent form, and they agreed to return after completion of treatment. Patients were diagnosed with primary hyperhidrosis, and they had intact skin at the level of the electrode application area. All of them were Romanian-speaking patients.

The exclusion criteria were as follows: a diagnosis of secondary hyperhidrosis; iontophoresis treatment in the last 6 months; and skin lesions at the level of the electrode application area. The contraindications for performing iontophoresis were skin lesions, infections, metal osteosynthesis materials in the area of electrode application, and epilepsy. Patients whose parents did not express their consent to participate and those with neuropsychiatric pathology, which may influence the responses, were excluded. Those who met the following criteria during the study were also excluded: withdrawal of consent, non-attendance at all 10 treatment sessions, and non-completion of the two questionnaires required for analysis.

The Hyperhidrosis Disease Severity Scale is a simple, single-item questionnaire designed to assess sweat tolerability and its interference with daily life. This measurement tool has four degrees for quantification of the severity of the impact of hyperhidrosis on the emotional state of the patient and the impairment of daily activities [9,17].

The HDSS is an important tool in defining treatment recommendations. A successful treatment is interpreted as an improvement from the fourth or third degree to the second or first degree, reflecting improvement in the quality of life of the patient [9]. 

All children began treatment on Monday and completed it for 5 consecutive days. Then, on Saturday and Sunday, they had time off and continued the following week with another 5 consecutive days, completing all 10 days on a Friday. Each treatment session lasted 10 min at a galvanic current intensity of 1 mA. Seven days after completing the treatment, on the following Friday, the children completed the Hyperhidrosis Disease Severity Scale again. All patients performed iontophoresis in the spring at a relatively constant temperature. No adverse reactions were reported.

**Figure 1:**
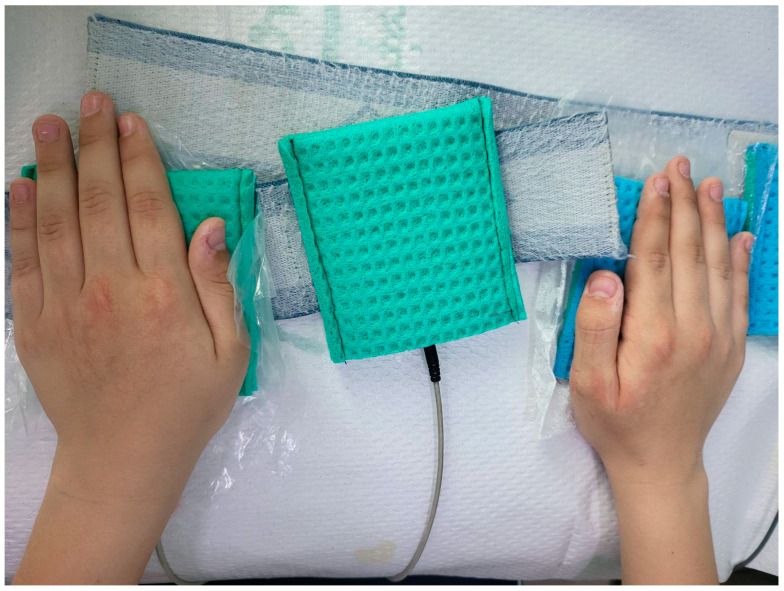
Application of surface electrodes (picture from a personal collection).

Ionogalvanization, or iontophoresis, is the method by which active substances are introduced in ionized form with the help of galvanic current, in our case water with baking soda. The chosen method of application is with surface electrodes (Figure 1) covered by a hydrophilic protective layer soaked in tap water mixed with baking soda.

The data obtained were statistically analyzed with XLSTAT 2024.

The aim of it was to raise an alarm signal about this underdiagnosed and undertreated disease, which has a major impact on the quality of life of children, adolescents, and their families.

## 3. Results

Patients in this study ranged from age 6–17 years (average 11.84 ± 2.89 years). There were 58 females (56.3%) and 45 males (43.7%), and the average age of females was slightly younger than males (11.5 vs. 12.2 years). The distribution of patient ages was: 6–8 years (13.6%), 9–11 years (35.0%), 12–14 years (26.2%), and 15–17 years (25.2%). 

### 3.1. T0—Before Starting Iontophoresis

In our study are included patients with scale values between 2 and 4, which correspond to a moderate or severe form of hyperhidrosis. The average value of the score is 2.95 ± 0.70. At T0, an HDSS score of 2 points was registered in 28 (27.18%) children, with 3 points in 52 (50.49%) children and 4 points in 23 (22.33%) children.

The girls who participated in this study have an average value of 2.96 ± 0.74, and the boys have an average value of 2.93 ± 0.65. 

### 3.2. T1—7 Days After the Completion of 10 Days of Iontophoresis

The mean value of the HDSS score at T1 is 1.92 ± 0.86. 

The girls who participated in this study have an average value of 1.89 ± 0.89, and the boys have an average value of 1.95 ± 0.82.

In Table 1, the average HDSS at T0 and T1 scores are analyzed according to the location of hyperhidrosis.

### 3.3. T0 versus T1 

An HDSS score of 1 point measured at T1 was recorded in 37 (35.92%) children, with 17 (45.94%) answers that decreased from the value of 2, 17 (45.94%) that decreased from the value of 3, and 3 (8.12%) that decreased from the value of 4.

An HDSS score of 2 points measured at T1 was recorded in 42 (40.78%) children, with 11 (26.19%) answers that remained at the value of 2, with 22 (52.38%) answers that decreased from the value of 3, and 9 answers (21.43%) that decreased from the value of 4.

An HDSS score of 3 points measured at T1 was recorded in 19 (18.45%) children with 13 (68.42%) answers that remained at the value of 3, and 6 (31.57%) answers that decreased from the value of 4.

An HDSS score of 4 points measured at T1 was recorded in 5 (4.85%) children that remained at the same value after treatment.

Treatment success was defined as an improvement in the HDSS score from 4/3 to 2/1 or from 2 to 1 [20]. Therefore, we can say that in our study the success of the treatment was recorded in 68 (66.02%) children, but a change in the score was recorded in 74 (71.84%) children. In the study carried out by Dagash et al. [21], the success rate was 84%, with a significant reduction in mean HDSS (pre 3.5 vs. post 2). In our study, the HDSS mean value decreased from 2.95 to 1.92.

The value t obs > t crit and the test parameter belong to the critical region, and the null hypothesis is thus rejected, being able to draw the conclusion that there is a statistical difference between the HDSS T0 and T1 values. Analysis of the correlation matrix between the HDSS values recorded at time T0 and those at time T1 indicated a moderate positive linear correlation, quantified by the Pearson correlation coefficient (r = 0.479), suggesting a statistically significant association between HDSS measurements at the two time points. The *p* value < 0.0001 supports the existence of a relationship between the HDSS values measured before and after the treatment, at a high level of significance.

The limitations of the study consist of the inclusion of only children with palmar, plantar, or palmo–plantar combination hyperhidrosis, because these areas can be treated by iontophoresis, and the lack of generalization of the results to other regions or populations because the study was conducted in a selected area, Galati County, a small town in southern Romania. The limitations of this study also include its focus on the short-term impact of iontophoresis and the lack of a control group against which to compare the results. Therefore, we believe that this opens up new opportunities for follow-up research, being complemented by multicenter studies carried out over a longer period of time to follow the long-term effects and also conducting comparative studies with a control group.

## 4. Discussion

### 4.1. Physiology and Pathophysiology

Sweat glands are of two major types: eccrine and apocrine. Apocrine glands are non-thermoregulatory, larger, and restricted to the axillae and the urogenital regions. They produce a viscid secretion that does not contribute to hyperhidrosis. Eccrine glands are tiny and very numerous, with approximately 3 million distributed throughout the skin [22]. Their distribution varies depending on the body area. The highest density is in the palm, the axilla, and the sole of the foot. Consisting of a simple coiled secretory portion deep inside the dermis, each gland is surrounded by a rich capillary plexus. A straight duct rises from the gland body through the epidermis, opening onto the surface of the skin. Their secretion mainly contains water with electrolytes, highlighting its thermoregulatory role, and also contains lactate, sodium, potassium, and urea for skin hydration and antimicrobial peptides to fight against skin infections and to control skin flora [22,23]. The hypothalamus controls the regulation of the volume and the rate of sweat production. It initiates measures to increase or decrease the body temperature after integrating and evaluating the sensory information related to the core body temperature. The secretion rate is elevated during emotional or physical stress, while in hyperhidrosis, the basal level of sweat secretion is increased, and there is an exaggerated response to normal stimuli [24]. Evaporation of sweat from the surface of the skin plays a critical role in thermoregulation [25].

### 4.2. Quality of Life Assessment

The skin is the most visible and prominent part of the body, playing a crucial role in how individuals are perceived, especially among young people [26,27]. Loneliness can be defined as the low quality of relationships or as the lack of socialization of a person due to the absence of intimacy, closeness, and emotional connection [28,29]. Teenagers with hyperhidrosis, like other skin problems, acne or psoriasis, for example, are known to have an increased level of loneliness [30]. Although skin diseases, such as acne vulgaris or hyperhidrosis, are not life-threatening, they can change appearance; therefore, they can affect daily activities, psychosocial status, quality of life, and relationships [28]. 

There is no commonly accepted amount of sweating to define hyperhidrosis. HH is often defined in qualitative terms. Abnormal sweating is defined as sweating that substantially interferes with an individual’s quality of life and affects his or her daily life. The focus in diagnosing hyperhidrosis is the subjective perception of the impact that sweat has on daily activities. It is imperative to evaluate the effects of hyperhidrosis on the patient’s quality of life and all the resulting daily life impairments. Impact on QoL is the most essential and valuable evaluation in hyperhidrosis [9]. Interpretation of hyperhidrosis and its impact is subjective, reflecting one’s own traits and feelings and bringing together various factors, such as mental, physical, and emotional wellbeing, medical health, education level, and cultural background [9,26].

Other than the HDSS, quality of life evaluation questionnaires for hyperhidrosis can be: *disease-specific*: HHIQ—Hyperhidrosis Impact Questionnaire, Keller Questionnaire, Campos Questionnaire; *dermatology-related*: DLQI—The Dermatology Life Quality Index, Skindex; and *general*: SF-36—Short Form questionnaire—36 items [9].

In 1995, a children’s version of this questionnaire was created, named The Children’s Dermatology Life Quality Index (CDLQI) [31,32]. This evaluates the quality of life of children over 4 years old with skin diseases and is available in both text and cartoon version. The children’s version also includes 10 questions, with the same grades [33].

The quality of life of children can be deeply affected by skin diseases, disrupting social relationships and families, and interfering with school, sport, and play. 

These measurements are necessary for the evaluation of new therapies or techniques or for clinical research. If the two versions of the questionnaire are compared, the adult one and the children’s one, questions 1, 2, 4, 5, 6, and 10 are the same. Adult questions about shopping, problems with partners, caring for the house or garden, or sexual difficulties were replaced with child-appropriate questions about friendships, sleep, and adverse comments. Adult problems relating to work are inappropriate for children, and this question was replaced with one relating to worries about school and holidays [31]. In 2003, a cartoon version of the CDLQI was developed to be more child-friendly. The animated character is a friendly dog, thus avoiding issues relating to age or to choosing a boy or girl. In this format, the original text is positioned near the colorful cartoons. The two types of questionnaires are equivalent, with the score remaining the same regardless of whether the written or the drawn version is used [34,35,36].

## 5. Conclusions

Our study explored the effect of iontophoresis on children with hyperhidrosis. The average HDSS score at T0 (2.95) was significantly reduced at T1 (after 10 treatments, to 1.89, *p* < 0.001). Treatment success (defined as a decrease in HDSS scores) was achieved in 68 (66%) of children, with a change in score in 75 (72%) of patients in this study. The intervention was well-tolerated by this pediatric cohort without reports of adverse effects. This study is limited by the patient sample size and the duration of the study, with a 10-day treatment effect observed and a lack of any longer-term data on the effect of this intervention.

## Figures and Tables

**Table 1:** HDSS T0 and T1 mean score in palmar, palmo–plantar, and plantar hyperhidrosis.

Statistic	HDSS—T0 Palmar	HDSS—T1 Palmar	HDSS—T0 Palmo–Plantar	HDSS—T1 Palmo–Plantar	HDSS—T0 Plantar	HDSS—T1 Plantar
No. of obs.	28	28	62	62	13	13
Minimum	2.000	1.000	2.000	1.000	2.000	1.000
Maximum	4.000	4.000	4.000	4.000	4.000	3.000
1st Quartile	3.000	1.000	2.250	1.000	2.000	1.000
Median	3.000	2.000	3.000	2.000	3.000	2.000
3rd Quartile	3.000	2.250	3.000	2.000	4.000	2.000
Mean	3.000	2.036	2.952	1.903	2.846	1.769
Variance (*n* − 1)	0.444	0.925	0.473	0.712	0.808	0.526
Standard deviation (*n* − 1)	0.667	0.962	0.688	0.844	0.899	0.725

## Data Availability

The data presented in this study are available on request from the corresponding author. The data are not publicly available due to ethical reasons.
